# Clarification of the Concept of *Ganoderma orbiforme* with High Morphological Plasticity

**DOI:** 10.1371/journal.pone.0098733

**Published:** 2014-05-29

**Authors:** Dong-Mei Wang, Sheng-Hua Wu, Yi-Jian Yao

**Affiliations:** 1 State Key Laboratory of Mycology, Institute of Microbiology, Chinese Academy of Sciences, Beijing, China; 2 Department of Botany, National Museum of Natural Science, Taichung, Taiwan, China; Ruhr-University Bochum, Germany

## Abstract

*Ganoderma* has been considered a very difficult genus among the polypores to classify and is currently in a state of taxonomic chaos. In a study of *Ganoderma* collections including numerous type specimens, we found that six species namely *G. cupreum*, *G. densizonatum*, *G. limushanense*, *G. mastoporum*, *G. orbiforme*, *G. subtornatum*, and records of *G. fornicatum* from Mainland China and Taiwan are very similar to one another in basidiocarp texture, pilear cuticle structure, context color, pore color and basidiospore characteristics. Further, we sequenced the nrDNA ITS region (ITS1 and ITS2) and partial mtDNA SSU region of the studied materials, and performed phylogenetic analyses based on these sequence data. The nrDNA ITS sequence analysis results show that the eight nrDNA ITS sequences derived from this study have single-nucleotide polymorphisms in ITS1 and/or ITS2 at inter- and intra-individual levels. In the nrDNA ITS phylogenetic trees, all the sequences from this study are grouped together with those of *G. cupreum* and *G. mastoporum* retrieved from GenBank to form a distinct clade. The mtDNA SSU sequence analysis results reveal that the five mtDNA SSU sequences derived from this study are clustered together with those of *G. cupreum* retrieved from GenBank and also form a distinct clade in the mtDNA SSU phylogenetic trees. Based on morphological and molecular data, we conclude that the studied taxa are conspecific. Among the names assigned to this species, *G. fornicatum* given to Asian collections has nomenclatural priority over the others. However, the type of *G. fornicatum* from Brazil is probably lost and a modern description based on the type lacks. The identification of the Asian collections to *G. fornicatum* therefore cannot be confirmed. To the best of our knowledge, *G. orbiforme* is the earliest valid name for use.

## Introduction

*Ganoderma* P. Karst. (*Ganodermataceae*, *Basidiomycetes*) is a widespread genus of wood decaying polypore fungi, with high species diversity in the tropics [1]. This genus is well-known for its high medicinal properties especially in East Asia [2], [3], [4], and also for its pathogenicity in South and Southeast Asia [5], [6] and East Asia [7]. Microscopically, *Ganoderma* is easily recognized by its ellipsoid to ovoid, umbonate, often truncate and double-walled basidiospores with yellow-brown echinulate to minutely reticulated inner layer.

However, the identification and circumscription of species within *Ganoderma* are unclear for various reasons. Species have been described only from a single collection or locality [8], or recognized based on environment- or development-dependent characters [9]. Type or authentic specimens lacking modern descriptions are lost, and controversial synonyms and nomenclatural combinations or misapplied names exist [8]. Diverse taxonomic criteria have been employed by various researchers [10]–[14] with a wide spectrum of morphological variability [15]. Currently, *Ganoderma* is in a state of taxonomic chaos as indicated by the fact that ca. one-third of 219 species proposed within the genus are synonyms and some of the remaining species still require further clarification [8], [9].

The internal transcribed spacer of nuclear ribosomal DNA (nrDNA ITS) composed of ITS1 and ITS2 is under low functional constraints and more variable than coding regions. During the last two decades, nrDNA ITS region has been widely used for inferring fungal species relationships, and selected as a standard marker for fungal DNA barcoding [16]. In 1995, Moncalvo et al. [17] noted that nrDNA ITS sequences can discriminate between most species of *Ganoderma*. Later on, nrDNA ITS sequencing has been widely applied to the identification and discrimination of *Ganoderma* species [18]–[20].

Hong et al. [21] revealed that the sequence from nearly complete mitochondrial small subunit ribosomal DNA (mtDNA SSU) has 3.3 times more information than nrDNA ITS sequences among the studied species of *Ganoderma*. Hong and Jung [22] inferred the phylogenetic relationship between species of *Ganoderma* based on nearly complete mtDNA SSU sequences. They concluded that both conserved domains and variable domains (V1–V9) of this studied fragment contained valuable phylogenetic information of *Ganoderma* species. In GenBank database, however, only the fragment of mtDNA SSU corresponding to the variable domains V3 to V 5 [22] has been widely sequenced.

To provide useful information for clarifying the taxonomic status of this genus, we have performed studies of type and voucher collections of *Ganoderma* species with an emphasis on Chinese materials in recent years. In this survey, we found that six species namely *G. cupreum* (Sacc.) Bres., *G. densizonatum* J.D. Zhao & X.Q. Zhang, *G. limushanense* J.D. Zhao & X.Q. Zhang, *G. mastoporum* (Lév.) Pat., *G. orbiforme* (Fr.) Ryvarden, *G. subtornatum* Murrill, and records of *G. fornicatum* (Fr.) Pat. from Mainland China and Taiwan are morphologically very similar to one another. Further, we sequenced the entire ITS nrDNA including the intervening 5.8S coding region, and partial mtDNA SSU corresponding to variable domains V3 to V5 [22] of the studied materials for phylogenetic analyses. The results derived from both morphological and molecular data are reported here.

## Materials and Methods

### Ethics statement

For field collections, no locations privately-owned or protected in any way were visited and therefore no permits are required. No endangered or protected species are involved.

### Morphological Study

Twenty dried herbarium materials and four freshly collected basidiocarps were subjected to careful morphological examinations in this study. The studied specimens have diverse geographical origins: China, Phillippines, Indonesia, Singapore (Asia) and Guinea (Africa). All the studied specimens are deposited at BPI, HMAS, TNM, TNS and UPS. Herbaria abbreviations follow Holmgren and Holmgren [23].

Morphological studies were performed as described [24], [25]. For observations of microscopic characters, 5% KOH was used as mounting medium except for cuticle structure. At least 20 basidiospores were measured from each mature specimen except for very scanty materials. The basidiospore size was measured both with and without the myxosporium based on those with collapsed apex, but only spore sizes with myxosporium were used for comparisons. The cuticle sections were taken from the mature pilear portion and mounted in Melzer's reagent for observations. Images and line drawings of cuticle structure and basidiospores were respectively prepared with the video system mounted on a Zeiss Axioskop microscope and the assistance of a camera lucida.

### DNA extraction, PCR amplification and DNA sequencing

Samples for DNA extraction were from dried specimens or subculture of living strains grown in 2% liquid malt extract medium. Total DNA was extracted by following the protocol provided previously [26], or the instructions of the Plant Genomic DNA Extraction Miniprep System (Viogene, Taiwan). The primer pairs ITS5(ITS1)/ITS4 and MS1/MS2 [27] were used for amplifying the entire nrDNA ITS and partial mtDNA SSU, respectively. The reaction components and conditions of PCR amplification were previously described [26], [28]. Double stranded DNA sequencing was performed in ABI 3100 or ABI 3730 DNA Analyzer.

### Sequence alignment and phylogenetic analysis

The sequences derived from this study (GenBank accession nos: JX840345–JX840352 (nrDNA ITS); KC581711–KC581712, KJ595577–KJ595579 (mtDNA SSU)) were compared with all the sequences of *Ganoderma* from the same molecular marker in GenBank and from Smith and Sivasithamparam [20] (whose nrDNA ITS sequences were not submitted to GenBank, but available from this publication). Sequences were first aligned by using Clustal X 1.83 [29] and then manually adjusted by using BioEdit 7.0.4.1 [30]. Based on the criteria described [26] and sequence availability from GenBank, 48 nrDNA ITS sequences and 22 mtDNA SSU sequences including outgroup taxon *Tomophagus* Murrill (and *Amauroderma* Murrill) were chosen after the initial analyses. When different submissions for the same material in GenBank occur, the sequence with higher quality was used for this study. For those materials whose mtDNA SSU sequences were chosen for further analysis, we also included them in the final analysis of nrDNA ITS sequences if available except for the strain of *G. tsugae* Murrill ATCC 64794. The nrDNA ITS and mtDNA SSU sequences of this strain were published by Park et al. [31] and Hong and Jung [22], respectively. We noticed that it is difficult to align the nrDNA ITS sequence of this strain (GenBank accession no. JQ675674) with the chosen nrDNA ITS sequences of *Ganoderma*. Further BLAST search revealed that it is a pollution sequence and was therefore excluded from our analysis. The details of all the chosen sequences are given in Table 1. The sequence alignment files were subjected to final analyses of maximum-parsimony (MP) in PAUP* 4.0b10 [32]. The analytical parameter preferences were specified as described [28]. Bootstrap analysis [33] was performed with 1000 replicates with random addition sequences to obtain estimates of the reliability of the nodes.

**Table 1:** Taxa used in this study and their DNA sequences accessed in GenBank or publication.

Original species name^a^	Specimen/strain^b^	Locality	nrDNA ITS^c^	mtDNA SSU^c^
*Amauroderma rude*	JMM ASP.1	Taiwan	X78753&X78774	–
Ganoderma adspersum*	CBS 351.74	Belgium	X78742&X78763	–
G. applanatum*	ATCC 44053	Japan	JQ520161	AH012391
G. australe*	UWA 108	Australia	AJ627590&AJ627591	–
*G. boninense* ^◊^	GR376	Unknown	–	FJ154775
*G. boninense* ^◊^	Unkown	Unknown	BD082757	–
*G. boninense* ^◊^	FA5035	Unknown	EU701010	–
*G. boninense* ^◊^	RS	Taiwan	X78749&X78770	–
*G. colossum*	CBS 268.88	USA	–	AF248337
*G. colossus*	CBS 216.36	Philippines	Z37071&Z37091	–
*G. cupreum*	KL16	India	FJ655466	–
*G. cupreum*	KR15	India	FJ655469	–
*G. cupreum*	DFP 4336	Australia	AJ627588&AJ627589	–
*G. cupreum*	DFP 3896	Australia	AJ627586&AJ627587	–
*G. cupreum*	SUT H1	Australia	AY569450	–
***G. cupreum***	HMAS 99399	Mainland China	JX840346	KC581711
***G. cupreum***	HMAS 130804	Mainland China	JX840345	KC581712
*G. cupreum*	GanoTK7	Cameroon	JN105702	JN105730
*G. cupreum*	GanoTK4	Cameroon	JN105701	JN105732
*G. formosanum* ^◊^	RSH 0109	Taiwan	X78752&X78773	–
***G. fornicatum***	BCRC 35374	Taiwan	JX840349	KJ595578
***G. fornicatum***	TNM-F0009926	Taiwan	JX840348	–
***G. fornicatum***	TNM-F0010592	Taiwan	JX840347	KJ595577
G. incrassatum*	DAR 73783	unknown	[18]	–
G. lobatum*	CBS 222.48	USA	X78740&X78761	AH012384
***G. lucidum*** **^◊^**	TNM-F0005258	Taiwan	EU021461	–
***G. lucidum*** **^◊^**	ATCC 64251	Taiwan	JQ520187	AF214475
***G. lucidum*** **^◊^**	CBS 270.81	France	–	AF214467
*G. lucidum* ^◊^	HMAS 86597	England, UK	AY884176	–
*G. mastoporum*	PM2/F-27198	Malaysia	JQ409361	–
***G. mastoporum***	TNM-F0018783	Mainland China	JX840352	KJ595579
***G. mastoporum***	TNM-F0018835	Mainland China	JX840351	–
***G. mastoporum***	TNM-F0018838	Mainland China	JX840350	–
*G. mastoporum*	CMU-HM1	Thailand	JN643730	–
*G. mastoporum*	GDGM 25720	Mainland China	JX195201	–
*G. meredithae* ^◊^	ATCC 64492	USA	JQ520190	AF248343
*G. meredithae* ^◊^	CBS 269.88	USA	–	AF248344
*G. orbiforme*	BCC22324	Thailand	JX997990	–
***G. oregonense*** **^◊^**	ATCC 46750	Canada	Z37061&Z37101	–
***G. oregonense*** **^◊^**	CBS 177.30	Canada	Z37060&Z37100	AF214471
***G. oregonense*** **^◊^**	CBS 264.88	USA	–	AF248346
G. philippii*	IMI 108700	Malaysia	AJ608714&AJ608715	–
*G. resinaceum* ^◊^	CBS 152.27	UK	JQ520200	AF214472
*G. ryvardense* ^◊^	HKAS 58053	Cameroon	HM138671	–
*G. ryvardense* ^◊^	HKAS58054	Cameroon	HM138672	–
*G. sinense* ^◊^	ZHANG 1734	Mainland China	Z37066&Z37103	–
*G. subamboinense* var. *laevisporum* ^◊^	ATCC 52419	Argentina	X78736&X78757	AF248349
*G. subamboinense* var. *laevisporum* ^◊^	ATCC 52420	Argentina	JQ520205	AF248348
***G. tornatum*** *	BAFC 2764	Argentina	AH008105	–
***G. tropicum*** **^◊^**	TNM-F0017073	Taiwan	EU021458	–
***G. tsugae*** **^◊^**	CBS 428.84	USA	X78735&X78756	–
*G. tsugae* ^◊^	ATCC 64794	USA	JQ675674	AF248350
***G. weberianum*** **^◊^**	CBS 219.36	Philippines	JQ520219	–
***G. weberianum*** **^◊^**	GanoTK06	Cameroon	JN105703	JN105721

a The original species name in bold indicates that the material is subjected to DNA sequencing in this study; ^*^ and ^◊^ respectively represent dull and laccate species which have been accepted worldwide.

b The basidiocarps from which DFP 4336 and DFP 3896 are derived were determined as *G. chalceum* by RL Steyaert [20]. Smith and Sivasithamparam redetermined these two associated isolates as *G. cupreum* based on the conspecificity between *G. cupreum* and *G. chalceum* [52] and the principle of nomenclatural priority [8].

c ‘–’ indicates that the sequence is unavailable for this study. The sequence of DAR 73783 can be retrieved only from publication [20]. The framed sequence accession no indicates a pollution sequence.

## Results

### Phylogenetic analysis

**Figure 1:**
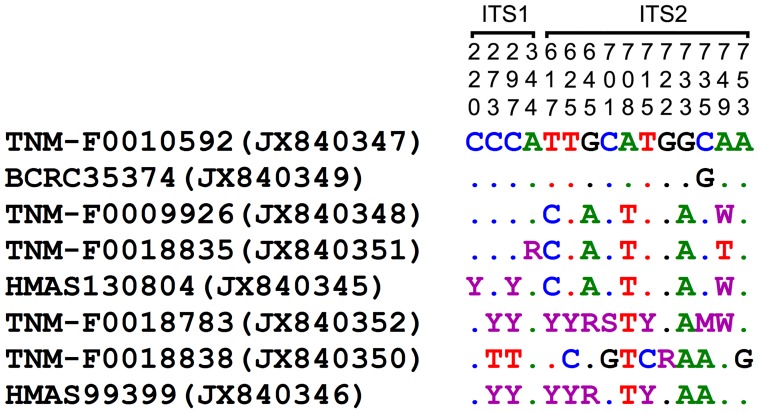
Variable sites in the ITS1 and ITS2 sequences of *G. orbiforme* from this study. Nucleotide positions (top) correspond to positions in the original alignment matrix for the phylogenetic analysis. Specimen/strain nos of taxa follow from Table 1 and are listed on the left along with GenBank accession nos in parentheses. Dot stands for ‘identity’ to the reference sequence of TNM-F0010592. M, R, S, W and Y stand for ‘AC’, ‘AG’, ‘CG’, ‘AT’ and ‘CT’, respectively.

The nrDNA ITS amplification delimited by the primer pairs ITS1/ITS4 and ITS5/ITS4 yields PCR products of ca. 650 bp and 670 bp long, respectively. All the eight nrDNA ITS sequences derived from this study (JX840345–JX840352, Table 1) differ from one another in one to 11 single-nucleotide substitution(s) in the combined ITS1 and ITS2 region. Six of them have intra-individual nrDNA ITS polymorphic site(s): one for each of TNM-F0009926, TNM-F0018835 and TNM-F0018838; three for HMAS 130804; six for HMAS 99399; nine for TNM-F0018783. The variations of the eight nrDNA ITS sequences at inter- and intra-individual levels are indicated in Figure 1.

The PCR product of mtDNA SSU amplified by using MS1/MS2 is ca. 580 bp long. Among the five mtDNA SSU sequences derived from this study (Table 1), three with different geographical origins (KJ595577–KJ595579) are identical to one another in both conserved domains and variable domains after excluding several ambiguous sites at both ends. The other two sequences differ from this shared sequence compositions by KJ595577–KJ595579 in a single-base transversion (KC581712) or in a single-base transition and two-base insertion (KC581711) within the range of alignment. The sequences of *G. cupreum* retrieved from GenBank (JN105730 and JN105732) are identical to one of the five sequences from this study (KC581711) in the hypervariable domain 4 [22], except for two transitions and two transversions (JN105730) and two more transversions (JN105732). Unlike the strain named *G. lucidum* (Curtis) P. Karst. ATCC 64251 with a more than 1500-bp intron (AF214475) [21], the mtDNA SSU sequences of *G. cupreum* have no intron.

The final alignments of the 48 nrDNA ITS and 22 mtDNA SSU sequences include 887 and 3610 positions, respectively. For nrDNA ITS, 430 sites are used for the MP analysis after excluding the conserved 18S rDNA, 28S rDNA and 5.8S rDNA regions. Among the sites included, 249 are constant, 58 are variable but parsimoniously uninformative, and 123 are parsimoniously informative. Totally 1475 most parsimonious trees (TL = 404, CI = 0.584, RI = 0.820) are generated. For mtDNA SSU, 2061 sites are used for the MP analysis after excluding the ambiguous sites at both ends. Of the included sites, 2008 are constant, 12 are variable but parsimoniously uninformative, and 41 are parsimoniously informative. A total of 266 most parsimonious trees (TL = 89, CI = 0.753, RI = 0.854) are obtained.

**Figure 2:**
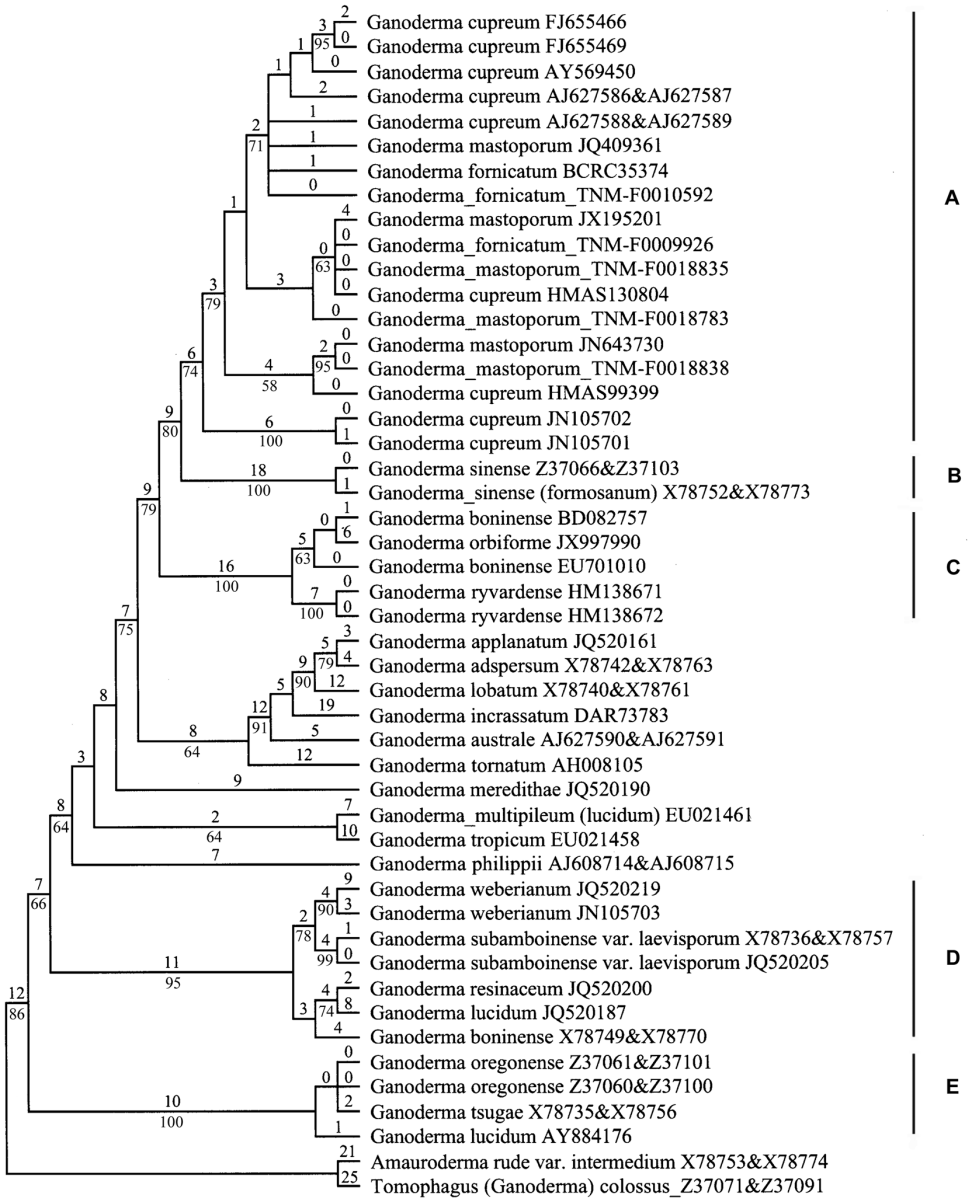
One of the 1475 most parsimonious trees derived from the nrDNA ITS sequence data. The upper and lower numerals at the nodes denote the number of estimated substitutions and proportions of bootstrap replicates, respectively. Only bootstrap values ≥50% are shown.

The 1475 nrDNA ITS trees are identical in topologies. One of them is shown in Figure 2 and five clades (A–E) are designated for the purpose of discussion. The other trees differ from Figure 2 mainly in the arrangement of taxa labeled *G. cupreum*, *G. fornicatum* and *G. mastoporum* within clade A. In Figure 2, all the materials of *G. cupreum*, *G. fornicatum* and *G. mastoporum* are grouped together with a moderate bootstrap support (Clade A, BS = 74%). Within this clade, the sequences with the same species name do not form subclades. The step changes among the sequences within clade A vary from zero to 19. *G. sinense* clade (Clade B, BS = 100%), and one clade composed of *G. boninense* (BD082757 and EU701010), *G. orbiforme* (JX997990) and *G. ryvardense* (Clade C, BS = 100%) served as the first and second sister clades of clade A, respectively. There are 24 and 31 step changes between clades A and B, and between clades A and C, respectively. In addition, one material labeled *G. boninense* (X78749&X78770) nests into the *G. resinaceum* complex clade (Clade D, BS = 95%) comprising *G. lucidum* (JQ520187), *G. resinaceum*, *G. subamboinense* var. *laevisporum* and *G. weberianum*. JQ520187 is not clustered into the *G. lucidum* complex clade (Clade E) where *G. lucidum* from the type locality belongs to (AY884176).

**Figure 3:**
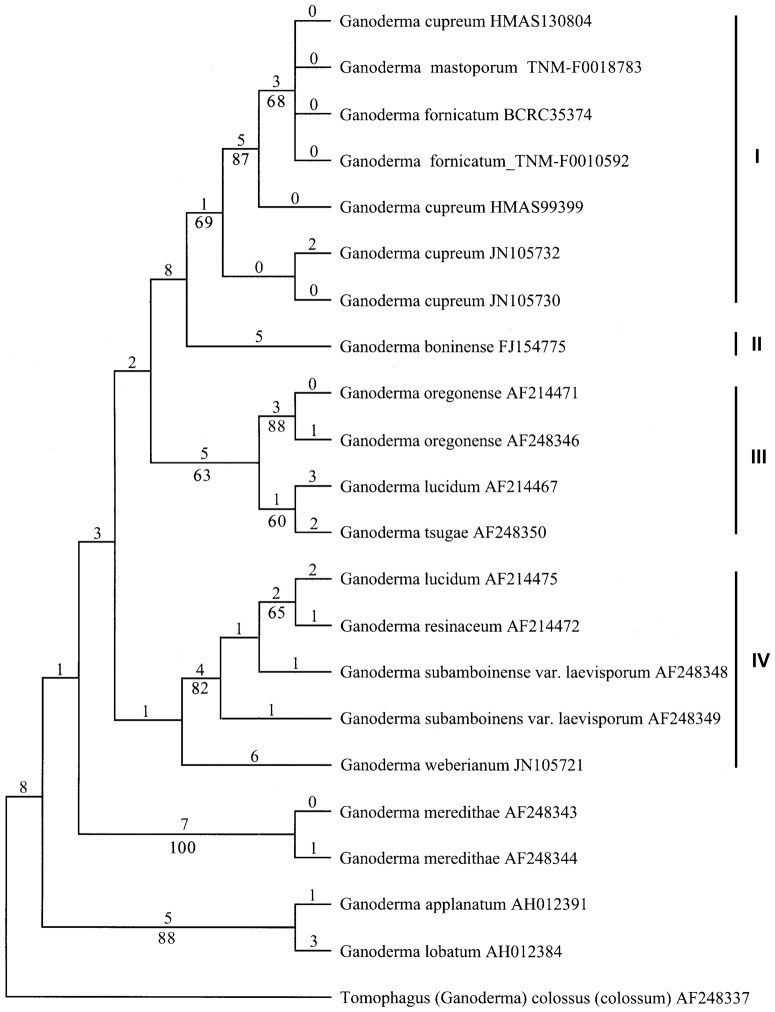
One of the 266 most parsimonious trees derived from the mtDNA SSU sequence data. The upper and lower numerals at the nodes denote the number of estimated substitutions and proportions of bootstrap replicates, respectively. Only bootstrap values ≥50% are shown.

One of the mtDNA SSU trees is shown in Figure 3, where the five sequences from this study originally labeled *G. cupreum*, *G. fornicatum* and *G. mastoporum* are clustered together with all the sequences of *G. cupreum* retrieved from GenBank (Clade I, BS = 69%). *G. boninense* (FJ154775, clade II) serves as the sister clade of clade I, but with a lower bootstrap support (BS<50%). Clades III and IV correspond to clades E and D in the nrDNA ITS tree (Figure 2), respectively. The other mtDNA SSU trees differ from Figure 3 mainly in taxa grouping within clade I, and the arrangement of *G. boninense* (Clade II) and of *G. weberianum* (Clade IV). In some trees, *G. boninense* is grouped with *G. applanatum* and *G. lobatum*, and *G. weberianum* is clustered with *G. meredithae*.

### Taxonomy

**Figure 4:**
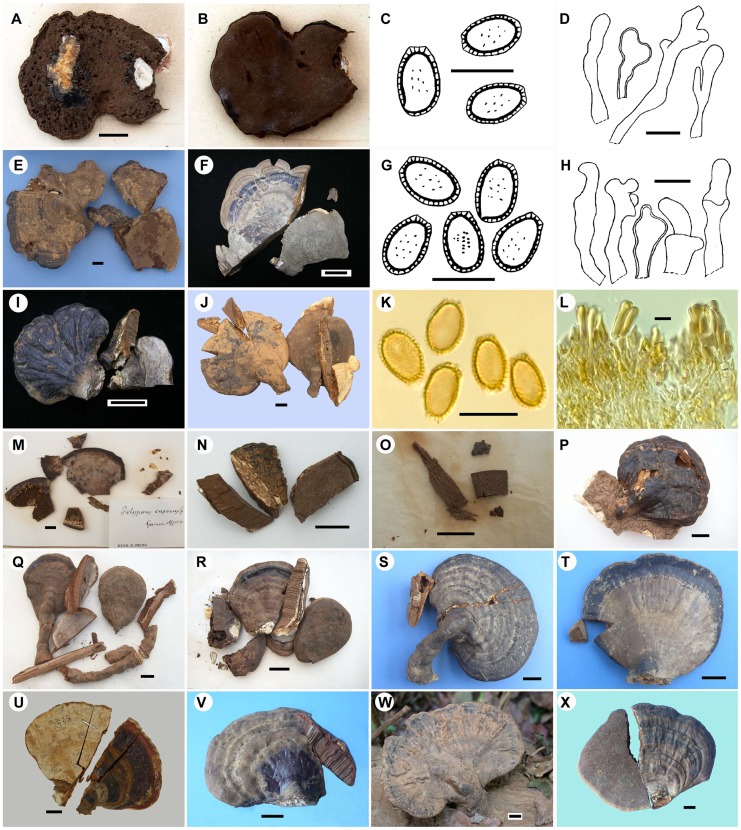
Morphology of *Ganoderma orbiforme*. **A–D.** Holotype of *G. orbiforme* in UPS (A. Basidiocarp upper surface, B. Basidiocarp lower surface, C. Basdiospores, D. Cuticle cells). **E.** HMAS 47610 originally as holotype of *G. densizonatum* (basidiocarps). **F–H.** TNM-F0010592 (G. Basdiospores, H. Cuticle cells). **I.** TNM-F0009926. **J–L.** HMAS 47065 originally as holotype of *G. limushanense* (K. Basidiospores, L. Cuticle structure). **M.** Collection originally as holotype of *G. cupreum* (Basidiocarp fragments). **N.** BPI-US0236899 originally as isotype of *G. subtornatum* (Basidiocarp fragments). **O.** BPI-US0236597 originally as isotype of *G. mastoporum* (Basidiocarp fragments). **P.** BPI-US0236596. **Q.** BPI-US0236598. **R.** BPI-US0236595. **S.** HMAS 37892. **T.** HMAS 38248. **U.** TNM-F0018783. **V.** HMAS 130804. **W.** HMAS 99392. **X.** HMAS 99399. Scale bars  = 1 cm in **A**, **E**, **F**, **I**, **J** and **M**-**X**;  = 10 µm in **C**, **D**, **G**, **H**, **K** and **L**.

***Ganoderma orbiforme*** (Fr.) Ryvarden [as ‘*orbiformum*’], Mycologia 92(1): 187 (2000). (Figure 4)

≡*Polyporus orbiformis* Fr., Epicr. Syst. Mycol. (Upsaliae): 463 (1838).

≡*Fomes orbiformis* (Fr.) Cooke, Grevillea 14(no. 69): 18 (1885).

 = *Polyporus mastoporus* Lév., Annls Sci. Nat., Bot., sér. 3 2: 182 (1844).

 = *Ganoderma mastoporum* (Lév.) Pat., Bull. Soc. Mycol. Fr. 5: 71 (1889).

 = *Polyporus cupreus* Fr., Nova Acta R. Soc. Scient. upsal., Ser. 3 1: 64 (1851).

 = *Ganoderma cupreum* (Sacc.) Bres., Ann. Mycol. 9: 268 (1911).

 = *Ganoderma subtornatum* Murrill, Bull. Torrey Bot. Club 34: 477 (1907).

 = *Ganoderma fornicatum* (Fr.) Pat. *sensu* Imazeki, Bull. Tokyo Sci. Mus. 1: 47 (1939)

and Zhao & Zhang, Flora Fungorum Sinicorum. 18: *Ganodermataceae*: 204 (2000).

 = *Ganoderma densizonatum* J.D. Zhao & X.Q. Zhang, Acta Mycol. Sin. 5(2): 86 (1986).

 = *Ganoderma limushanense* J.D. Zhao & X.Q. Zhang, Acta Mycol. Sin. 5(4): 219 (1986).

***Basidiocarps*** annual to perennial, sessile (usually with a slightly or distinctly contracted base) to stipitate, woody. ***Pilei*** 2.5–9.5×3.5–12.7 cm, up to 2.8 cm thick at the base, subreniform, flabelliform, suborbicular or spathulate inoutline; upper surface orange yellow to orange red when young, becoming purplish red, purplish black, black to grayish brown or grayish black when old, sometimes with alternating orange red, purplish red to black zones or alternating grayish brown to grayish black zones, weakly to strongly laccate, partly laccate or dull, distinctly concentrically sulcate or not, slightly or distinctly radially rugose or not, usually with a deposit of pale brown basidiospores; margin obtuse, rounded or slightly lobate, yellowish white or concolorous with the pileus. ***Pore surface*** straw yellow when young, becoming purplish brown when old; tubes up to 1.2 cm long in total, pale brown or smoky brown, without context layer between tube layers; pores circular or subcircular, 5–7(−10)/mm, (30−)50–80(−130) µm diam., (70−)90–120(−160) µm dissepiments. ***Stipe*** when present, 0.8–6×0.5–2.3 cm, dorsally lateral or horizontally lateral, cylindrical, concolorous with the pileus. ***Context*** up to 2.4 cm thick, pale brown to reddish brown tinged with whitened streaks or patches near the cutis, with black crustose lines, corky to woody; generative hyphae 2–5.5 µm diam., colorless, thin-walled, with clamp-connections; skeletal hyphae 3.5–7 µm diam., yellowish brown to reddish brown in KOH, dextrinoid; binding hyphae 1.5–3.5 µm diam., colorless, thick-walled, much-branched. ***Basidiospores*** (8.5−)9.5–12.5×(5.5−)6–7(7.5) µm (with myxosporium), (7.5−)8.5–10×(4.5−)5.5–6.5 µm (without myxosporium), ellipsoid to ovoid, brown, with a brown eusporium bearing fine, short and slightly distinct echinulae. ***Cuticle*** in the laccate portion 15–80×3–10.5 µm, hymeniodermic, composed of clavate or apically acanthus-like branched cells, inamyloid, dextrinoid to weakly or strongly amyloid.

*Specimens examined*: CHINA, Mainland China, Hainan, Bawangling, Yajia Forest Farm, alt. 800 m, on rotten wood, 18 April 1977, S-J Han et al. *HN777* (HMAS 47610; holotype of *G. densizonatum*); Bawangling, Yajia Forest Farm, alt. 1000 m, on rotten wood, 20 April 1977, S-J Han et al. *HN885* (HMAS 38248; originally determined as *G. mastoporum*); Jianfengling, on fallen wood, 17 December 2003, G-Z Zhao *wdm61* (HMAS 130804; originally determined as *G. cupreum*); Jianfengling, on fallen wood of a broad-leaved tree, 17 December 2003, D-M Wang *wdm62R* (HMAS 130816; originally determined as *G. cupreum*); Jianfengling, alt. 800 m, on fallen wood of a broadleaf tree, May 2004, X-Q Zhang and L-D Guo *3938-1* (TNM-F0018783; originally determined as *G. mastoporum*); Jianfengling, alt. 800 m, on wood, 7 August 2004, H-Q Chen *4679-1* (TNM-F0018835; originally determined as *G. mastoporum*) and H-Q Chen *4722-1* (TNM-F0018838; originally determined as *G. mastoporum*); Limushan, on rotten wood, 3 April 1977, S-J Han et al. *HN272* (HMAS 37892; originally determined as *G. fornicatum*); Limushan, on rotten wood, April 1977, S-J Han et al. *HN464* (HMAS 47065; holotype of *G. limushanense*); Wuzhishan, on fallen wood of a broad-leaved tree, 14 December 2003, D-M Wang *wdm54* (HMAS 99399; originally determined as *G. cupreum*); Wuzhishan, on fallen wood of a broad-leaved tree, 14 December 2003, D-M Wang *wdm55* (HMAS 99392; originally determined as *G. cupreum*). Taiwan, Nantou, Lienhuachih, 23°56′N, 120°53′E, alt. 700 m, on fallen trunk of angiosperm, 15 June 1999, S-Z Chen *Chen 890* (TNM-F0009926; originally determined as *G. fornicatum*); Pingtung, Chufengshan, 22°04′N, 120°51′E, alt. 300 m, on wood, 13 November 1995, S-H Chang *CWN 01363* (TNM-F0004443; originally determined as *G. fornicatum*); Chufengshan, 22°04′N, 120°51′E, alt. 300 m, on rotten wood, 10 February 1998, C-C Wen *CWN 02880* (TNM-F0010815; originally determined as *G. lucidum*); Nanjenshan, 22°05′N, 120°50′E, alt. 250 m, on rotten wood, 12 April 1998, C-C Wen *CWN 03065* (TNM-F0010876; originally determined as *G. lucidum*); Taipei, Urai, December 1908, S. Kusano, *201.259* (TNS-F-201259; cited as *G. fornicatum* [34]); Taitung, Orchid Island, on the way to Tienchih, 22°01′N, 121°34′E, alt. 50 m, on wood of angiosperm, 24 October 1999, S-Z Chen *Chen 946* (TNM-F0010592; first identified as *G. densizonatum*, later determined as *G. fornicatum*). –INDONESIA, Irian Jaya (formerly Dutch New Guinea), Siwi in arfak Mts., on dead trees and wood, 26–29 June 1926, O.A. Reinking *#283A* (BPI-US0236595; originally determined as *G. mastoporum*); Momi, Teluk Cederawasih (formerly Geelvink Bay), on dead trees and wood, 30 June–2 July 1926, O.A. Reinking *#124A* (BPI-US0236598; originally determined as *G. mastoporum*). – GUINEA, A. Afzelius (UPS; holotype of *G. cupreum*); A. Afzelius (UPS (F-09957) 163110; holotype of *G. orbiforme*). –PHILIPPINES, Luzon, Lamao River, on a decayed trunk, November 1903, R.S. Williams (BPI-US0236899; labeled as ‘Isotype’ of *G. subtornatum* on the identification card); Mt. Maquiling, on dead wood, November 1920, O.A. Reinking *#10907* (BPI-US0236596; originally determined as *G. mastoporum*). –SINGAPORE, comm. Gaudichaud (BPI-US0236597; isotype of *G. mastoporum*).

## Discussion

Moncalvo et al. [17] stated that high nucleotide divergence is usually observed in the nrDNA ITS2 region in recently diverged taxa of *Ganoderma*. Similarly, all or most nucleotide variations (71%–83%) are located in the nrDNA ITS2 region of the materials sequenced in this study with reference to the sequence of TNM-F0010592 (JX840347, Figure 1). In addition, we noticed that six of the eight materials subjected to nrDNA ITS sequencing in this study have intra-individual single-nucleotide polymorphisms ranging from one to nine sites (Figure 1). This intra-individual heterogeneity phenomenon is observed in most of the materials sequenced in this study, but it does not interfere with direct DNA sequencing and phylogenetic relationship between species studied. All the nrDNA ITS sequences obtained in this study are clustered into the same monophyletic clade in the phylogenetic tree (Clade A, Figure 2). Further, the five mtDNA SSU sequences derived from this study are very similar to the sequences of *G. cupreum* retrieved from GenBank in base compositions after excluding ambiguous sites at both ends. They are grouped together to form a monophyletic clade in the mtDNA SSU phylogenetic tree (Clade I, Figure 3). Based on a combined analysis of these molecular results and related morphological data, we conclude that all of the materials examined in this study are conspecific.

Among the names assigned to this species, *G. fornicatum* given to Asian collections has nomenclatural priority over the others. Fries [35] first published this species as ‘*Polyporus* (*Pleurotus*) *fornicatus*’ based on collections from Brazil. Patouillard [36] transferred it to *Ganoderma* after examining collections from French Guiana (formerly Guyane). In East Asia, *G. fornicatum* has been accepted by Imazeki [34], Zhao and Zhang [37] and Wang and Wu [38]. Unfortunately, the type of *G. fornicatum* from Brazil cannot be located and is suspected to be lost [8], [39], [40]. A modern description of this species based on type lacks. Therefore, the identity of Asian collections referring to this name cannot be confirmed in morphology. Besides, no DNA sequence is obtained from the South American material. It is hard to say that both Asian and South American collections are molecularly conspecific. Currently, it is better to use other earlier name whose species identity can be verified.

*Ganoderma orbiforme* originally described as ‘*Polyporus orbiformis*’ from Guinea [41], is the second earliest name applied to the fungus. Ryvarden [42] studied the holotype of this species deposited in UPS and published it as a new combination under *Ganoderma*. We also examined the morphology of the same type specimen in UPS. It appears that this specimen has been attacked by insects and the pilear upper surface and context layer are destroyed (Figure 4A). The remaining material reveals that *G. orbiforme* has a rigid basidiocarp, purplish black laccate crust, purplish brown pore surface, brown tube layer, ellipsoid or ovoid, mostly truncate basidiospores with fine and short echinulae (10–12×6.5–7.5 µm), and cuticle composed of strongly amyloid, clavate cells usually with several irregular lobes or protuberances (30–80×3–10.5 µm). Based on examining the morphology of this holotype and other collections cited in this study, we conclude that *G. orbiforme* can be suitably applied to the species representing clade A (Figure 2) and clade I (Figure 3). The reliable features to recognize *G. orbiforme* are its rigid basidiocarp with a weakly to strongly laccate, partly laccate or dull pileus, variably brown context, ellipsoid to ovoid basidiospores with fine and short echinulae, and purplish brown pore surface at maturity. Besides, brown context with intermingled wood-colored hyphae and black crustose lines is often observed in collections of *G. orbiforme*, e.g. BPI-US0236595, BPI-US0236899, HMAS 47065. In addition, one material labeled *G. orbiforme* in GenBank (JX997990) is not grouped with real *G. orbiforme* in our nrDNA ITS sequence analysis (Clade A, Figure 2). By referring to Isaka et al. [43], this material (JX997990) has elongated basidiospores measuring 10–12.5×4–5 µm. This spore feature distinctly differs from that of real *G. orbiforme*, but is very similar to that of *G. boninense* Pat. as will be discussed further below.

Our study reveals that the collections of *G. orbiforme* exhibit great variability in morphology (Figure 4), although they conform to the above diagnostic features very well. The basidiocarp varies from sessile but with a contracted base form (e.g. HMAS 38248) to dorsally laterally stipitate (e.g. HMAS 47610) or horizontally laterally stipitate form (e.g. TNM-F0010592). The pilear shape is variable: subreniform (e.g. HMAS 37892), flabelliform (e.g. TNM-F0009926), suborbicular (e.g. HMAS 99399), spathulate (e.g. BPI-US0236598). The pilear color varies from orange yellow to orange red when young, becoming purplish red, purplish black, black to grayish brown or grayish black with age, as clearly revealed by collections at different stages of development (e.g. TNM-F0018783, TNM-F0010592, HMAS 37892). Besides, the pilear upper surface ranges from laccate (e.g. TNM-F0009926), partly laccate (e.g. BPI-US0236595) to dull (e.g. the dull basidiocarp of HMAS 47065), and also varies in ornamentations: distinctly concentrically sulcate (e.g. HMAS 47610), weakly concentrically sulcate (e.g. BPI-US0236596), distinctly radially rugose (e.g. TNM-F0009926), or slightly radially rugose (e.g. HMAS 130804). The pore surface color ranges from straw yellow in younger specimens (e.g. TNM-F0018783) to purplish brown in older specimens (e.g. holotype of *G. orbiforme* (UPS)). The cuticle structure is composed of usually irregularly clavate cells in the laccate portion (e.g. holotype of *G. orbiforme*, UPS), but these clavate cells disappear in the dull portion (e.g. HMAS 47065). The reaction of cuticle cells in Melzer's reagent ranges from inamyloid, dextrinoid to variably amyloid (e.g. TNM-F0010592).

Some of the above-mentioned variable features have been considered diagnostic of *Ganoderma* species by different taxonomists. Lloyd [39] recognized *G. mastoporum* (as ‘*Ganodermus mastoporus*’) as a distinct species with a lateral stipe. We also observed dorsally-lateral stipes in the authentic materials of *G. mastoporum*, e.g. BPI-US0236596 (Figure 4P). Stipe development varies with different growing environment as discussed previously [9], [44]. Zhao and Zhang [45] stated that *G. limushanense* differs from *G. subtornatum*; the former species has a non-laccate or partly laccate pileus with a yellowish brown to brown or near black upper surface, brown or fusco-brown context with intermingled white hyphae and crustose layers, while the latter species has the black laccate cuticle, duplex context with white upper layer and chestnut brown lower layer, and absence of crustose layer. Based on our observations, however, pilear color and laccate shine vary in *G. orbiforme*. The intermingled white hyphae can become patches with age and form a duplex context layer in some collections of *G. orbiforme*. The grayish black crustose layers exist in the type collection of *G. subtornatum* BPI-US0236899. It should be mentioned that the characteristic of crustose layer (presence or absence) has been often regarded as a diagnostic feature of *Ganoderma* species by researchers, but its taxonomic value requires further confirmation. Zhao and Zhang [37] have previsously identified that *G. densizonatum* differs from *G. limushanense* in cuticle structure, but we observe clavate or irregularly clavate cells in laccate portions of both type collections HMAS 47610 and HMAS 47065. The laccate cuticle structure of HMAS 47610 is presented in Figure 4L. Our study suggests that caution should be taken while using variable morphological features for the circumscription and identification of *Ganoderma* species.

*Ganoderma subtornatum* was first described from Philippines by Murrill [46] based on three collections. After examining type and authentic specimens, Steyaert [47] concluded that they represented different species. For one collection cited in Murrill's publication [46], Steyaert [47] found two basidiocarps viz. ‘R.S. Williams, November 1903’ numbered 153 marked ‘type’ in Herbarium NY and further designated one of them as lectotype of *G. subtornatum* and the other as a new species *G. lamaoense* Steyaert. In the protologue, however, Murrill [46] did not assign any number to the collection of *G. subtornatum*. Further, Steyaert's statement that the lectotype of *G. subtornatum* has a duplex context: chamois above and chestnut below and the holotype of *G. lamaoense* was collected at alt. 150 m and has a blackish brown (Ridgway) pileus [47], is inconsistent with the information given by Murrill [46]. In this study, we examined one type collection of *G. subtornatum* deposited at BPI which is also marked ‘R.S. Williams, November 1903’. Other information such as ‘isotype’, ‘Philippines, Luzon, Lamao River’, ‘Trunk decayed’, and ‘Herb. James R. Weir 23352’ are also provided for this collection. This BPI type includes only basidiocarp fragments (Figure 4N), but with entire sections. Its observable features conform to the protologue of *G. subtornatum* very well. As for the other two collections cited in Murrill's publication [46], Steyaert determined them as *G. chalceum* (Cooke) Steyaert or as a new species *G. leytense* Steyaert [47]. In fact, Steyaert suggested the conspecificity between *G. chalceum* and *G. cupreum*, but mistook *G. chalceum* as the valid name [46]. Two cultures DFP 4336 and DFP 3896 (Table 1) isolated from the basidiocarps determined as *G. chalceum* by RL Steyaert [20] are clustered into the real *G. orbiforme* clade in our nrDNA ITS phylogenetic analysis (Clade A, Figure 2) and further supports the conspecificity between *G. cupreum* and *G. orbiforme*. Corner [14] questioned the discrimination of *G. lamaoense* and *G. leytense* from *G. chalceum*. We cannot exclude *G. lamaoense* and *G. leytense* from being probable synonyms of *G. orbiforme* with further reference to their protologues [47].

As is well-known, cuticle with laccate shine or not has been used for discriminating between two groups *Ganoderma* (Type species: *G. lucidum*) and *Elfvingia* P. Karst (Type species: *G. applanatum* (Pers.) Pat.). However, the grouping of *G. orbiforme* challenges this taxonomic criterion. Ryvarden [42] treated *G. orbifrome* as a laccate species of *Ganoderma*. Moncalvo and Ryvarden [8] summarized the groupings of the synonyms of *G. orbiforme* treated in this study: *G. cupreum* and *G. subtornatum* (*Ganoderma* group), *G. densizonatum* and *G. limushanense* (*Elfvingia* group), and *G. mastoporum* (*Ganoderma* group? or *Elfvingia* group?). As revealed in this study, laccate, partially laccate and dull cuticles coexist in collection(s) of *G. orbiforme*. That's why it is impossible to group *G. orbiforme* with certainty or controversial groupings have been caused only based on this taxonomic criterion of pilear characteristic. Corner [14] noted that the cuticle of *G. mastoporum* is intermediate for hymenioderma is defective and clavate cells disappear in the mature crust as confirmed in our study. Currently, laccate *Ganoderma* and non-laccate *Elfvingia* have been widely accepted as two subgenera of *Ganoderma*. Smith and Sivasithamparam [20] stated that the phylogeny inferring from the nrDNA ITS sequences of five species of *Ganoderma* from Australia also supported the retention of these two subgenera. However, we found that the universally accepted laccate species and dull species (Table 1) are interspersed throughout the clades and do not form two distinct groups (laccate and dull) in the phylogenetic tree (Figure 2). Our study indicates that the grouping based on the presence or absence of laccate shine is easy to use in practice, but it does not reflect true phylogenetic relationship.

This study reveals that five described species and new records of Mainland China and Taiwan are conspecific with *G. orbiforme*. Accordingly, our study expands the distribution of *G. orbiforme* from Guinea to China, India, Thailand, Philippines, Malaysia, Singapore and Australia. By referring to Imazaki [34] and Smith and Sivasithamparam [20], [48], *G. orbiforme* is also distributed in Japan, Palau, Pohnpei (formerly Ponape), New Guinea and Solomon Islands. As mentioned above, the South American species *G. fornicatum* has been used to name Asian collections of *G. orbiforme*. The probably lost type of *G. fornicatum* and lack of associated modern description refrain us from further confirmation. But the protologue of *G. fornicatum* [35] and related morphological descriptions provided by Patouillard [36] and Lloyd [39] are not incongruent with the morphology of *G. orbiforme*. We cannot exclude the possibility that *G. fornicatum* might be an earlier name for *G. orbiforme*. Lloyd [39] commented that *G. fornicatum* is frequent in the type locality (Brazil). In recent years, however, no information of *G. fornicatum* has been reported from the type locality or adjacent regions. The confirmation of molecular data from South American materials will be desirable for further research. We believe that there have still been more species names ever assigned to *G. orbiforme*, e.g. twelve species names given to *G. mastoporum* in the Philippine collections [49], members of the *G. chalceum* complex [8], [14]. A further study is required for clarifying the geographical distribution of *G. orbiforme*.

It should be mentioned that a Taiwanese strain labeled *G. fornicatum* RSH 0814 has been often chosen for phylogenetic studies of *Ganoderma* [18], [20]. We examined the morphology of its cultivated fruiting body and compared its nrDNA ITS sequence with those of related taxa (data not shown). Both morphological and molecular data suggest that this strain should be redetermined as *G. tropicum* (Jungh.) Bres., a species also distributed in the tropics and subtropics [47], [50]. Besides, the strain ATCC 64251 from Taiwan is also found misidentified by referring to our nrDNA ITS and mtDNA SSU sequence analyses (Figures 2 and 3).

*Ganoderma sinense* J.D. Zhao, L.W. Hsu & X.Q. Zhang was published as a new species by Zhao et al. [51], based on collections from Hainan. This species is morphologically similar to *G. orbiforme* by having a purplish black to black laccate pileus, uniformly brown context or with whitish streaks or patches near the cuticle, a dorsally lateral or lateral stipe and a subtropical-tropical distribution [24]. However, *G. sinense* can be easily recognized from *G. orbiforme* by having an erect stipe, cuticle structure always composed of typically clavate cells, and ovoid basidiospores with few, long and thick echinulae [24]. Further, our nrDNA ITS sequence analysis also separates *G. sinense* and *G. orbiforme* into two clades (Clades A and B, Figure 2). Therefore, we recognize *G. sinense* as a species distinct from *G. orbiforme*.

*Ganoderma boninense* was first described based on the collection from Bonin Island [36]. Steyaert [52] provided a full description of this species with basidiospore size range 8.5–9.7– (10.9)–13.0–13.5×4.5–5.4–(5.9)–6.3–7.5 µm based on collections (including lectotype) from East Asia, Southeast Asia and Australia. After studying the lectotype (K, PC) and several Australian collections of *G. boninense* (including specimens studied by RL Steyaert), Smith and Sivasithamparam [48] concluded that the characteristic of elongated basidiospores (8.2–13.5×5–8.6 µm) is the main feature to separate *G. boninense* from *G. cupreum*, a synonym of *G. orbiforme* suggested in this study. In our nrDNA ITS sequence analysis (Figure 2), three materials labeled *G. boninense* are not grouped together; one (X78749&X78770) is clustered into the *G. resinaceum* complex clade (Clade D), and the other two (BD082757, EU701010) nest into Clade C. We studied authentic collections of *G. resinaceum* Boud. from Europe (type locality), and reveals that Clade D is the clade where *G. resinaceum* belongs to and *G. resinaceum* does not have elongated basidiospores (data not shown). Obviously, the material of *G. boninense* (X78749&X78770) has been misidentified as also discussed in Utomo et al. [53]. The other two materials labeled *G. boninense* (BD082757, EU701010) are grouped together with the material (JX997990, mistaken as *G. orbiforme*) with elongated basidiospores as mentioned above. In our mtDNA SSU sequence analysis, *G. boninense* is also separated from *G. orbiforme* (Clades I and II, Figure 3). It seems that both molecular results support this taxonomic criterion to separate *G. boninense* from *G. cupreum* (a synonym of *G. orbiforme*) concluded by Smith and Sivasithamparam [48]. If these materials with elongated basidiospores represent real *G. boninense*, our study does not support the treatment of *G. boninense* as a synonym of *G. orbiforme* [42].

Clade C also includes *G. ryvardense* R.K. Tonjock & A.M. Mih, a recently described pathogenic species to oil palm from Cameroon [54]. *G. ryvardense* shows a closer relationship to ‘*G. boninense*’ with elongated basidiospores (Clade C, Figure 2), but its spore size ((9–)10–13(–14)×(5–)6–8 µm) and spore morphology [54] are very similar to those of *G. orbiforme*. The relationships among *G. ryvardense*, *G. boninense* and *G. orbiforme* require further clarification.
